# Predictors of Nodal Metastasis in Cutaneous Head and Neck Cancers

**DOI:** 10.1007/s11912-022-01249-5

**Published:** 2022-04-08

**Authors:** Albert Y. Han, Maie A. St. John

**Affiliations:** 1 Department of Head and Neck Surgery, University of California Los Angeles (UCLA), Los Angeles, CA USA; 2 UCLA Head and Neck Cancer Program, UCLA Medical Center, 10833 Le Conte Ave, 62-132 CHS, Los Angeles, CA 90095 USA; 3 Jonsson Comprehensive Cancer Center, UCLA Medical Center, Los Angeles, CA USA

**Keywords:** Cutaneous cancer, Squamous cell carcinoma, Melanoma, Regional metastasis, Parotid metastasis

## Abstract

**Purpose of Review:**

The complex and varied drainage patterns in the head and neck present a challenge in the regional control of cutaneous neoplasms. Lymph node involvement significantly diminishes survival, often warranting more aggressive treatment. Here, we review the risk factors associated with lymphatic metastasis, in the context of the evolving role of sentinel lymph node biopsy.

**Recent Findings:**

In cutaneous head and neck melanomas, tumor thickness, age, size, mitosis, ulceration, and specific histology have been associated with lymph node metastasis (LNM). In head and neck cutaneous squamous cell carcinomas, tumor thickness, size, perineural invasion, and immunosuppression are all risk factors for nodal metastasis. The risk factors for lymph node involvement in Merkel cell carcinoma are not yet fully defined, but emerging evidence indicates that tumor thickness and size may be  associated with regional metastasis.

**Summary:**

The specific factors that predict a greater risk of LNM for cutaneous head and neck cancers generally include depth of invasion, tumor size, mitotic rate, ulceration, immunosuppression, and other histopathological factors.

## Introduction

Cutaneous neoplasms are clinically categorized into melanoma and non-melanoma skin cancers. Non-melanoma skin cancers include cutaneous squamous cell carcinoma (cSCC), basal cell carcinoma (BCC), Merkel cell carcinoma (MCC), and other less common tumors including sarcomas and adnexal tumors. BCC typically undergoes localized slow growth and rarely metastasizes, but cSCC, melanoma, and other malignancies often spread to regional and distant sites, which can significantly impact the clinical course of the disease and patient outcomes. The exact incidence of cSCC is unknown as cSCCs are often excluded in national tumor registries. However, a recent estimate indicated a global prevalence of 3.1 million cases of malignant melanoma and 2.2 million cases of cSCC in 2015 [1]. Despite the relatively small surface area of the head and neck region, approximately 60-70% of cSCCs [2] and 20% of cutaneous melanomas [3] arise in the head and neck.

**Fig. 1:**
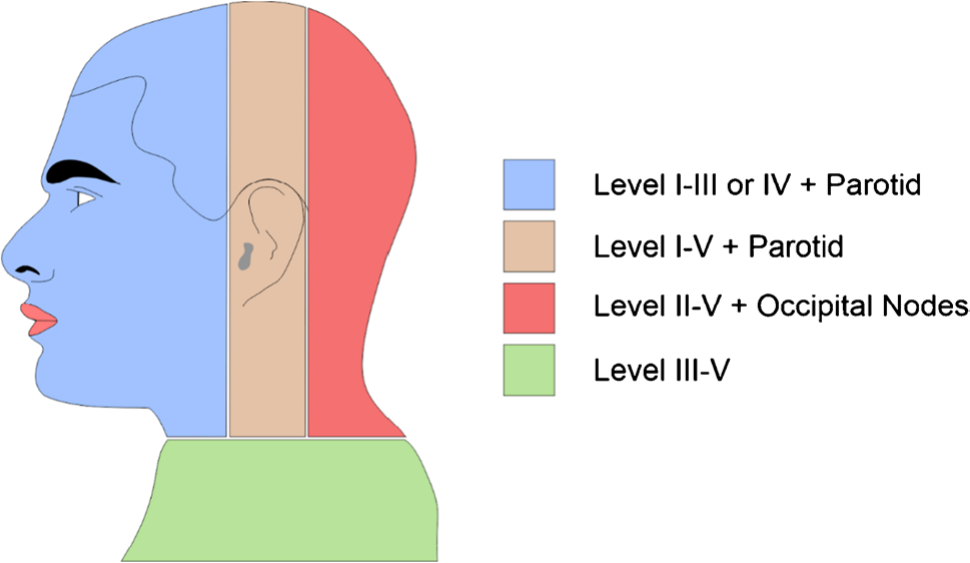
Predicted pattern of metastasis of head and neck cutaneous melanoma proposed by O’Brien et al. The orange area represents the “watershed area” from which unpredictable drainage can occur ( Adapted from O’Brien et al. *American Journal of Surgery*) [4]

Cutaneous cancers of the head and neck often spread via the lymphatic system toward the neck, frequently involving the intraparotid lymph nodes depending on the location of the primary tumor. The drainage pattern in the head and neck assumes a general division between the anterior and posterior skin zones with a proposed watershed zone in between (Fig. 1) [4]. The posterior head and neck regions drain to the occipital, postauricular, cervical level V, and supraclavicular fossa. In contrast, the anterior head and neck regions drain to the anterior cervical chains, as well as the parotid and preauricular nodes [5]. Approximately 20–40% of head and neck neoplasms spread to lymph nodes outside of clinically predicted levels [6, 7]. When using a sentinel lymph node biopsy (SLNB) in the head and neck or trunk regions, preoperative lymphoscintigraphy or SPECT/CT is recommended to guide the location of interval (in-transit) nodes that might harbor disease [8]. Although not yet the standard of care, the literature supports the use of SPECT/CT as it has been found to increase the SLN yield, resulting in a greater ability to detect metastatic involvement [9].

## Cutaneous Squamous Cell Carcinoma of the Head and Neck

The prognosis for patients with cSCC of the head and neck (cSCCHN) is excellent when diagnosed early. However, a subset of these patients develops lymph node metastasis (LNM) and ultimately experiences poorer outcomes. LNM develops in 5% of patients after resection of the primary lesion [10]. If found at presentation or after treatment, LNM is associated with a higher 5-year mortality [11]. Involvement of the lymph nodes also increases the likelihood of recurrence to approximately 51% and decreases the 3-year disease-specific survival to 52%, even with adjuvant treatment [12]. Therefore, understanding the risk factors for metastasis in cSCCHN is critical for early identification of patients who need more aggressive, often multimodal, management.

### Depth of Invasion

Tumor depth of invasion (DOI) has been consistently reported as a risk factor for metastasis whether measured in Breslow thickness or histological depth [13]. The relative risk is higher for patients with tumors with a DOI cutoff of > 2 mm [14]. In one recent study, no metastasis was observed for superficial lesions with DOIs of less than 2 mm [10]. Tumor invasion beyond subcutaneous fat was associated with nodal metastasis (subhazard ratio 7.2) [15]. In a prediction model proposed by Wermker et al. [16], tumor depth and invasion of cartilage were two of the four indicators, along with recurrence number and grade, that accurately identified patients with cSCC of the ear who might benefit from neck dissection.

### Tumor Size

A tumor size greater than 20 mm was associated with disease progression, including regional metastasis [13]. For the current eighth edition staging system developed by the American Joint Committee on Cancer (AJCC), the primary tumor staging is determined by the dimensions of the tumor: T1 (2 cm or less in diameter) and T2 (greater than 2 cm but less than or equal to 4 cm). Tumor size is often associated with LNMs and worse survival with discrete cutoffs (e.g. greater than 20 mm in the greatest dimension) or as a continuous variable [17, 18, 19, 20]. Tumor size was also an independent predictor of nodal metastasis (> 20 mm) with a HR of 2.22 [10]. In a large study of 6,000 patients in New Zealand, tumor size as a continuous was a prognosticator of LNM with HR 1.41 (*p* < 0.0001) [21]. Alternative measurements of the tumor size, such as tumor volume greater than 2,500 mm, were significantly associated with LNMs as well [22].

### Subsite

Certain sites of the head and neck appear to be associated more with LNMs. During development, the face develops via integration of embryological processes that form planes of fusion. Tumors spread more readily between these facial zones, also known as *embryological fusion planes*. Mohs and Panje et al. defined a high-risk H-shaped “face mask” area for BCC and cSCCHN that includes the auricle, preauricular region, infraorbital area, nasolabial fold, and sublabial area [23, 24, 25]. While the impact of the embryonic fusion planes has been challenged, certain sites such as the ears, cheeks/temples, and lips have been found to be associated with nodal disease in multinomial logistic regression analyses [21, 26•]. The total risk for cSCCHN LNM of the auricle has been cited as 15.5%, which is the highest in all cSCC subsites [16].

### Perineural Invasion and Angiolymphatic Invasion

Histological features, such as perineural invasion (PNI) and poor histological differentiation, are significant predictors of cSCCHN LNM. In the study of the New Zealand population, PNI and poor histological differentiation were independent predictors of LNM with hazard ratios of 5.29 and 4.26, respectively [21]. A recent case series of 212 patients also confirmed PNI as a factor associated with nodal disease; however, poor histological differentiation was only associated with recurrence, not nodal metastasis [26•]. Wermker et al. included tumor grade as one of the variables in a prediction model that had the highest effect size with recurrence number and was significantly associated with LNM [16]. In recent studies examining positive SLNBs of cSCCHN, angiolymphatic invasion was significantly associated with the presence of nodal metastasis [27•].

### Immunocompromised Patients

In solid organ transplant recipients (SOTRs), cSCC is the most common skin malignancy. Skin cancers in SOTRs exhibit a much more aggressive clinical course. In a case series of 153 patients, SOTRs had an increase in the number of primary cancers, deep tissue involvement, PNI, LVI, and recurrence [28]. In these patients, the risk of nodal metastasis was an approximate 3.5 fold increase over immunocompetent controls. Immunosuppression was found to be an independent predictor of nodal metastasis (RR 4.32) in a larger multivariate analysis [10]. In a multi-institutional study, immunosuppressed patients with cSCCHN (including SOTRs and other etiologies) had significantly lower 2-year locoregional recurrence-free survival (47.3% vs. 86.1%) and progression-free survival (38.7% vs. 71.6%) [29].

### Implications of Intraparotid Lymph Nodes on Occult Neck Disease

cSCCHN can drain to the intraparotid lymph nodes as the first echelon node, depending on the location of the primary tumor. Of patients with a clinically positive parotid node (P + disease), 22.5–35% had occult neck metastasis, most commonly at level II [30, 31]. It is yet unclear whether parotid node involvement alters the overall prognosis. For this reason, the eighth edition AJCC Staging Manual does not differentiate parotid lymph node or regional LNM in N staging [32]. Furthermore, the extent of parotidectomy and neck dissection in the setting of cSCCHN parotid metastasis continues to be debated [33]. NCCN guidelines recommend superficial parotidectomy and ipsilateral neck dissection if clinical or pathologic parotid nodes are present [34].

### Cutaneous Squamous Cell Carcinomaof the Head and Neck Summary

In patients with tumors with high-risk features, occult nodal metastasis must be considered. High-risk features include recurrent disease, DOI > 2 mm, tumor size > 20 mm, poorly differentiated histology, location in the facial mask H region, PNI, angiolymphatic invasion, and immunosuppression. Concurrent lymph node dissection and parotidectomy must be considered in these patients. Given the potential for late relapses and regional metastases, patients who receive such treatments must undergo close surveillance. The role of SLNBs continues to take shape, as the surgical and histological methods are becoming more standardized [27•].

## Cutaneous head and neck melanoma

In the year 2020 alone, an estimated 100,350 new cases of melanoma were diagnosed, with 6,850 new deaths [35]. Cutaneous head and neck melanoma (CHNM) most frequently involves males in their 60 s [36]. The 10-year survival rate for scalp and neck melanoma is approximately 60%, with other subsites such as the ear, face, and eyelid having a better prognosis at 70%, 80%, and 90%, respectively [37]. Occult LNM occurs in 15–20% of clinically negative necks in CHNM [3]. Biopsy-driven assessment of the regional lymph nodes is key in the staging process for CHNM [38••]. The NCCN guidelines recommend considering SLNB for T1b melanoma (Breslow depth < 0.8 mm with ulceration or 0.8–1.0 mm with or without ulceration) or T1a lesions with Breslow depth < 0.8 mm and with other high-risk features [39]. The management of the neck has been controversial with early pioneers supporting an elective and complete neck dissection [40]. However, subsequent trials, including MSLT-II, failed to demonstrate a clear survival benefit with immediate completion neck dissection compared to a more conservative ultrasound surveillance after a positive SLNB [38••]. Indeed, in Faries et al., only a fraction (17%) of the included patients had melanoma in the head and neck area, and additional studies are needed to capture indications in which upfront neck dissection may be useful in CHNM. Understanding the predictors of LNM is critical as positive LNM carries prognostic and therapeutic implications.

### Tumor Thickness

The tumor thickness is associated with nodal metastasis and survival in CHNM. The eighth edition AJCC Staging Manual incorporated tumor thickness cutoff at 0.8 mm and ulceration to differentiate T1a from T1b, as ulceration was demonstrated to be a better prognosticator of melanoma specific survival (MSS) than mitotic rate [32]. Positive SLN occurs infrequently (< 5%) in 11s less than 0.8 mm thick; however, this number is higher (5–12%) in primary melanomas with thicknesses of 0.8 to 1.0 mm [32]. Large cancer database studies corroborate these findings and add that greater tumor depth is associated with a greater likelihood of LNM. In a National Cancer Database (NCDB) study of CHNM patients, a depth of 1 to 2 mm was associated with an odds ratio (OR) of 1.83, and a depth greater than 2 mm was associated with an OR of 2.88 compared to lymph node involvement in cases of tumor depth <1 mm [42•].

### Age

Increasing age was a negative predictor of overall survival and was negatively associated with lymph node involvement [42•]. For this reason, Yalamanchi et al. suggest discussing risks and benefits of SLNB in patients > 70 years of age and thickness < 1.0 mm. Indeed, patients older than 75 years with thin melanomas (1.01–1.49 mm) demonstrated less than 5% of SLN positivity [43]. The current NCCN guidelines recommend discussing and offering SLNB for older patients. [39].

### Subsite

In a NCDB database study, the face, scalp, and neck, as opposed to the lip and external ear, were found to be associated with an increased risk of LNM [42•]. Within the head and neck region, melanomas of the scalp had significantly higher nodal involvement compared to melanomas of the other parts of the head and neck. Furthermore, scalp location was an independent prognosticator for MSS even when controlling for Breslow thickness, T stage, ulceration, and lymph node positivity [44]. Indeed, melanoma of the scalp and neck had a 53% higher risk of mortality compared to that of the face [36]. Melanomas of the face provided better prognoses than that of the scalp, ear, and neck [45]. However, the subsite was not included in the eighth edition of AJCC Staging Manual due to variability in how the subsites were classified [32].

### Mitosis, Ulceration, and Histology

The mitotic rate is no longer used in the T staging criterion of the eighth edition of the AJCC Staging Manual. However, the *Melanoma Expert Panel* continues to recommend the assessment and reporting of the mitotic rate, as it may be important in alternative prognostic models [32]. A previous study demonstrated an association between increased mitotic rate and SLN positivity [46]. In a NCDB study, mitotic rate was strongly associated with lymph node status in thin melanomas (≤ 1.00 mm; including head and neck, trunk, and extremities) [47•]. In a separate NCDB study that focused on head and neck melanomas, this association between mitoses and LNM was confirmed (OR 1.44), along with an association with ulceration (OR 1.57) [42•]. The presence of microsatellites also significantly increased SLN positivity for all thin 1.00 mm cutaneous melanomas [48]. Histological subtype may have an impact on LNM as well. Single-institution cohort and SEER database studies demonstrated rare SLNB positivity (0% and 3.69%, respectively) in desmoplastic melanomas [49, 50]. However, conflicting evidence does exist in the literature, perhaps due to a lack of standardized criteria defining pure desmoplastic melanoma [39, 51].

### Non-sentinel Lymph Node Involvement

The involvement of non-sentinel lymph nodes is a risk factor for false-negative SLNB. In a study that enrolled 387 CHNM patients, SLNB of CHNM followed by complete neck dissection resulted in a non-sentinel lymph node (NSLN) positivity rate of 22% [52•]. In the same study, the size of tumor deposit in the SLN > 0.2 mm (*p* = 0.05) was a predictor of positive NSLN. Multicenter studies that focused on trunk and extremity melanomas revealed that NSLN positivity in 11 patients increased with the number of SLNs identified on LNM (> 3 SLNs = 24%), thicker depth, and trunk/head and neck location [53, 54, 55]. Among CHNM patients, 25% of patients who received SLNB demonstrated drainage to multiple basins, but the presence of multiple basin was not associated with increased SLN positivity or survival [56].

Multicenter Selective Lymphadenectomy Trial I (MSLT-I) provided definitive evidence that SLNB combined with immediate lymphadenectomy provides increased disease-free-survival among patients with intermediate thick (1.20–3.50 mm) and thick (> 3.50 mm) melanomas [57••]. The follow-up study, MSLT-II, revealed that an immediate completion lymphadenectomy after positive SLNB increased the rate of regional disease control and provided prognostic information but did not increase MSS among patients with positive sentinel-node metastases [38••]. NSLN metastases were found in 11.5% of the cohort, and this was an independent predictor recurrence (HR 1.78). At this time, a critical gap in the literature exists on high-risk CHNM patients who may benefit from completion neck dissection. As both MSLT studies only had a minority representation (13–18%) of CHNM patients, large prospective, randomized studies are required to understand the therapeutic utility of completion lymph node dissection for positive SLNB in CHNM.

### Implications of Intraparotid Lymph Nodes on Occult Neck Disease

No study to date has demonstrated survival advantage with elective parotidectomy in clinically negative parotid involvement. However, in a setting of clinically or microscopically positive intraparotid lymph nodes, a superficial parotidectomy and neck dissection are recommended by NCCN guidelines [39]. The incidence of occult parotid lymph node involvement in CHNM patients with cervical lymph node disease has been reported at 16.1–25% [58, 59]. For this reason, if a patient has high-risk features for relapse and metastasis, parotidectomy should be considered at the time of primary surgery.

### Consideration for SLNB in Patients Who Already Received a Prior Wide Local Excision for CHNM

Surgical removal of cutaneous neoplasms can disrupt lymphatic channels. The ablative process eliminates the precise location of the primary tumor for accurate tracer injection and can disrupt the local lymphatic drainage patterns. Previous studies have suggested an increased probability of regional metastasis after local advancement flaps [60]. Furthermore, this can result in difficulty identifying the true SLNs and may lead to a false negative result. A recent retrospective review of 391 patients with CHNM who received wide local excisions revealed that the sentinel lymph nodes can be successfully identified in all patients who had a prior wide local excision [61]. Although SLNB after excision may be feasible, concurrent SLNB and wide local excision is recommended to reduce the morbidity of multiple surgeries [62].

### CHNM Summary

SLN status serves as the most critical prognostic information in risk stratification and therapeutic planning in patients with CHNM. Risk factors for positive SLN include young age, scalp as the subsite, mitotic activity, ulceration, histology, and parotid involvement. The standard of care for regional management after a positive SLNB recently shifted to nodal basin ultrasound surveillance with adjuvant medical therapy as necessary as opposed to completion neck dissection and parotidectomy [63]. Future studies should delineate the high-risk patients who might benefit from completion neck dissection after a positive SLNB.

## Merkel Cell Carcinoma

MCC is a rare cutaneous neuroendocrine tumor that predominantly presents in the head and neck. The cornerstone of MCC management is surgery with negative margins, followed by adjuvant treatment. Occult nodal metastasis in MCC is common and presents in approximately 30% of patients, even with small tumor size [64]. Although a published report indicated that nodal evaluation might be avoided for tumors less than 1 cm in size [65], surmounting evidence reports the contrary, in that MCCs of all tumor sizes may harbor nodal metastases [66]. In particular, subsequent studies have demonstrated that MCC patients with tumor sizes < 1 cm still had positive SLNBs approximately 20–30% of the time [67, 68•, 69]. For this reason, SLNB has become an integral part of the management for draining the nodal basin and thus incorporated in the latest NCCN guidelines [70]. In these cases, an appropriate immunopanel, including CK20 and TTF-1, on the SLNB specimen can contribute additional information [70].

Clinicopathological features associated with positive SLN include increasing tumor thickness and infiltrative tumor growth pattern [66, 71]. An increasing mitotic rate [66] and lymphovascular invasion, as well as tumor-infiltrating lymphocytes were predictors of a positive SLN [72]. Although these factors are supported in the literature, some series such as the Mayo case series concluded a different conclusion that no tumor patient characteristics could predict SLN positivity [73].

The SLNB result has shown to be a significant prognosticator of survival [70]. The 3-year overall survival rate was significantly higher at 88% with a negative SLNB compared to 57% with a positive SLNB [67]. For this reason, completion lymph node dissection continues to be recommended [74].

## Future Directions

Despite the impressive strides being made in predicting patients with occult LNM, several gaps remain in the management of patients with cutaneous head and neck cancers. Studies are ongoing to define genetic and molecular biomarkers that can provide insight into whether aggressive transformations have occurred that predispose patients to regional and distant metastasis. In a recent translational study, a combined prediction model for SLNB positivity that incorporated gene expression signatures (e.g., glia-derived nexin, growth differentiation factor 15, and others) outperformed the use of clinicopathologic features alone [75].

Given the unique developmental anatomy and complex—sometimes described as ambiguous—lymphatic drainage pattern and head and neck cutaneous neoplasms likely need to be studied separately from truncal and extremity melanomas. Large prospective trials that guide melanoma management have often excluded or only included a small portion of CHNM patients (e.g., MSLT-I and MSLT-II). Future studies should focus exclusively on head and neck cutaneous neoplasms or allow for a greater representation in landmark trials. With increased understanding of the unique behavior of head and neck cutaneous neoplasms and their genetic and molecular markers, we will be able to determine the patient-specific risks of metastatic potential and thus offer more personalized treatment and precision surgery.

## Conclusions

SLNB has become a cornerstone in the assessment of the lymph node basin for staging and therapeutic planning in melanoma and MCC. The management of cSCCHN continues to evolve toward embracing SLNB. The role of completion neck dissection after positive SLNB in CHNM continues to be evaluated. The specific factors that predict a greater risk of LNM for cutaneous head and neck cancers generally include *depth of invasion, tumor size, mitotic rate, ulceration, immunosuppression, and other histopathological factors*. The limited data on CHNM warrants additional prospective trials to assess the therapeutic benefit of SLNB and the utility of completion lymph node dissection in high-risk patients. An evolving understanding of molecular and genetic biomarkers should be included in patient risk stratification, as more reliable markers under standardized processing become available. We can then offer our patients more personalized treatment and precision surgery to allow for better outcomes.
